# On the usage of average Hausdorff distance for segmentation performance assessment: hidden error when used for ranking

**DOI:** 10.1186/s41747-020-00200-2

**Published:** 2021-01-21

**Authors:** Orhun Utku Aydin, Abdel Aziz Taha, Adam Hilbert, Ahmed A. Khalil, Ivana Galinovic, Jochen B. Fiebach, Dietmar Frey, Vince Istvan Madai

**Affiliations:** 1 CLAIM - Charité Lab for Artificial Intelligence in Medicine, Charité Universitätsmedizin Berlin, Berlin, Germany; 2 Research Studio Data Science, Research Studios Austria, Salzburg, Austria; 3 Centre for Stroke Research Berlin, Charité Universitätsmedizin Berlin, Berlin, Germany; 4 Department of Neurology, Max Planck Institute for Human Cognitive and Brain Sciences, Leipzig, Germany; 5 Mind, Brain, Body Institute, Berlin School of Mind and Brain, Humboldt-Universität Berlin, Berlin, Germany; 6 School of Computing and Digital Technology, Faculty of Computing, Engineering and the Built Environment, Birmingham City University, Birmingham, UK

**Keywords:** Average Hausdorff distance, Cerebral angiography, Cerebral arteries, Image processing (computer-assisted)

## Abstract

Average Hausdorff distance is a widely used performance measure to calculate the distance between two point sets. In medical image segmentation, it is used to compare ground truth images with segmentations allowing their ranking. We identified, however, ranking errors of average Hausdorff distance making it less suitable for applications in segmentation performance assessment. To mitigate this error, we present a modified calculation of this performance measure that we have coined “balanced average Hausdorff distance”. To simulate segmentations for ranking, we manually created non-overlapping segmentation errors common in magnetic resonance angiography cerebral vessel segmentation as our use-case. Adding the created errors consecutively and randomly to the ground truth, we created sets of simulated segmentations with increasing number of errors. Each set of simulated segmentations was ranked using both performance measures. We calculated the Kendall rank correlation coefficient between the segmentation ranking and the number of errors in each simulated segmentation. The rankings produced by balanced average Hausdorff distance had a significantly higher median correlation (1.00) than those by average Hausdorff distance (0.89). In 200 total rankings, the former misranked 52 whilst the latter misranked 179 segmentations. Balanced average Hausdorff distance is more suitable for rankings and quality assessment of segmentations than average Hausdorff distance.

## Key points

- Average Hausdorff distance has a hidden error when used to rank medical image segmentations.
- Balanced average Hausdorff distance alleviates the ranking error of average Hausdorff distance.
- Balanced average Hausdorff distance should be used to rank medical image segmentations.

## Background

The average Hausdorff distance is a widely used performance measure to calculate the distance between two point sets. In medical image segmentation, it is used to compare ground truth images with segmentation results and allows ranking different segmentation results. Average Hausdorff distance has been applied to assess performance of various applications including brain tumour segmentation [1], cerebral vessel segmentation [2, 3], temporal bone segmentation [4], segmentation of the extracranial facial nerve [5], tumour volume delineation [6], colorectal liver metastases segmentation [7], prostate cancer lesion segmentation [8] and pylorus tracking on ultrasound images [9].

Average Hausdorff distance is especially recommended for segmentation tasks with complex boundaries and small thin segments such as cerebral vessel segmentation [10]. In comparison to other performance measures such as the Dice coefficient, average Hausdorff distance has the advantage that it takes voxel localisation into consideration. Unlike the Hausdorff distance that quantifies the largest segmentation error, average Hausdorff distance takes all distances of point pairs between two segmentations into account.

In this work, we show, however, that a ranking error in the usage of average Hausdorff distance makes it less suitable for segmentation performance assessment and ranking. We also present a new modified performance measure, coined *balanced average Hausdorff distance* to alleviate the ranking error.

## Methods

### Average Hausdorff distance

The average Hausdorff distance between two finite point sets *X* and *Y* is defined in eq. 1.

1 $$ {d}_{AHD}\left(X,Y\right)=\left(\frac{1}{X}\sum \limits_{x\in X}\underset{y\in Y}{\mathit{\min}}\;d\left(x,y\right)+\frac{1}{Y}\sum \limits_{y\in Y}\underset{x\in X}{\min}\;d\left(x,y\right)\right)/2 $$

The directed average Hausdorff distance from point set *X* to *Y* is given by the sum of all minimum distances from all points from point set *X* to *Y* divided by the number of points in *X*. Average Hausdorff distance can be calculated as the mean of the directed average Hausdorff distance from *X* to *Y* and directed average Hausdorff distance from *Y* to *X*.

In the medical image segmentation domain, the point sets *X* and *Y* refer to the voxels of the ground truth and the segmentation, respectively. The average Hausdorff distance between the voxel sets of ground truth and segmentation can be calculated in millimeters or voxels. Equation 1 can be written in a more simplified way as follows:

2 $$ Average\kern0.17em Hausdorff\kern0.17em distance=\left(\frac{GtoS}{G}+\frac{StoG}{S}\right)/2 $$

where *GtoS* is the directed average Hausdorff distance from ground truth to segmentation, *StoG* is the directed average Hausdorff distance from segmentation to ground truth, *G* is the number of voxels in the ground truth, and *S* is the number of voxels in the segmentation.

### Balanced average Hausdorff distance

Since each of the segmentations to be ranked is compared with the ground truth, the ranking error of average Hausdorff distance stems from the division by *S* which differs from one segmentation to the other depending on the number of voxels in each segmentation. The modified calculation is shown in eq. (3). Here, *StoG* is divided by *G*, which is constant for all segmentations.

This newly proposed performance measure is coined balanced average Hausdorff distance.

3 $$ Balanced\kern0.17em average\kern0.17em Hausdorff\kern0.17em distance=\left(\frac{GtoS}{G}+\frac{StoG}{G}\right)/2 $$

### Data

Time-of-flight magnetic resonance angiography images of 10 patients from the 1000Plus study were randomly selected. The 1000Plus study was carried out with approval from the local Ethics Committee of Charité University Hospital Berlin (EA4/026/08). Details about the study have been previously published [11]. The only inclusion criterion was no occlusion in any vessel segments constituting the circle of Willis. To create the ground truth of the cerebral arterial vessels, three-dimensional time-of-flight magnetic resonance angiography images were pre-segmented using a U-net-based deep learning framework and manually corrected by OUA and VIM using ITK-Snap [12] as described by Livne et al. [2].

### Error simulation

**Fig. 1:**
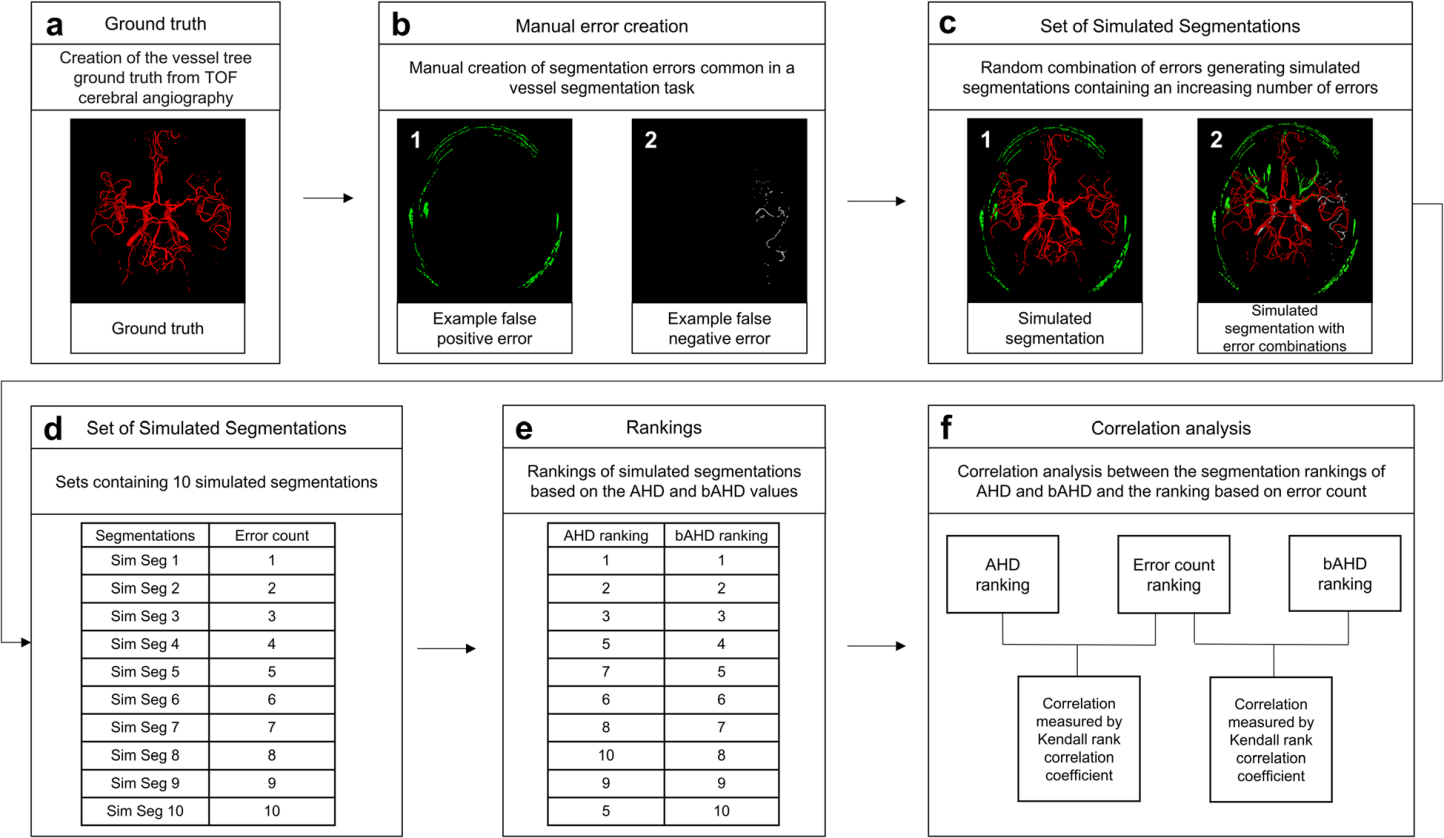
Flow chart of the error simulation framework and correlation analysis. **a** The ground truth was created using a U-Net deep learning architecture and subsequently manually corrected. **b** The voxels in the errors are added or subtracted from the ground truth depending on whether the error is a false positive or false negative error. **b1** Error introducing false positive voxels (green) in the skull area. **b2** Error in which false-negative voxels (white) in the M3 segment of the middle cerebral artery are missing. **c1** False-positive voxels in the skull area are added to the ground truth to create a simulated segmentation. **c2** The error simulation framework allows the random combination of manually created errors to create simulated segmentations containing multiple errors. This simulated segmentation was created by combining seven errors. **d** The ten simulated segmentations in the set have an increasing number of errors. **e** The simulated segmentations are ranked from best to worst using the average Hausdorff distance and balanced average Hausdorff distance values, respectively. **f** Lastly, the correlation between the rankings are measured by the Kendall rank correlation coefficient. The process is repeated using 20 sets of simulations for each patient

In order to explore the properties of AHD and bAHD more systematically for quality assessment of cerebral vessel segmentations, an error simulation framework was developed. To simulate segmentations for ranking, a set of 55 non-overlapping segmentation errors common in a vessel segmentation task were manually created. These errors included, for example, oversegmentation and undersegmentation of various vessel segments, false positively labelled other anatomical structures and omitted parts of the vessel tree. Of these 55 errors, one random error was added to the ground truth, the image was saved, and this process was successively repeated 9 times by adding each time a new random error to the resulting image and saving each image. The end result was a set of 10 simulated segmentation results with an increasing number of errors in a random combination. Twenty such sets were created for each of the ten patients. An illustration of the error simulation framework can be found in Fig. 1. The error simulation framework was programmed in Python (version 3.6.8); specifically, image manipulation was performed with the NiBabel package (version 2.3.0).

### Segmentation ranking

Average Hausdorff distance and the proposed balanced average Hausdorff distance were evaluated for their capability to rank the above generated segmentation sets. Each segmentation was evaluated against the ground truth using each of the two performance measures, yielding an average distance value measured in voxels. Each set of simulated segmentations was ranked using the two performance measures, where the best segmentation result got rank 1 and the lowest rank 10. Here, an ideal performance measure should have a perfect correlation between the produced ranking of simulated segmentations with the increasing number of errors as the next simulated segmentation has at all times an additional error compared to the previous segmentation.

### Statistical analysis

Kendall’s tau correlation coefficient for ordinal rankings was calculated between the segmentation rankings of the two performance measures and the number of errors for each simulated segmentation set. Kendall’s tau correlation coefficient measures the similarity of the orderings of the data when the data is ranked using two different approaches. The coefficient has the value 1 in case of a perfect agreement of two rankings and -1 in case of perfect disagreement. The median of Kendall’s tau correlation coefficients of the 20 ranking sets per patient was reported. For each patient, the two-sided Wilcoxon signed-rank test was performed to calculate whether the improvement of Kendall’s tau coefficient was statistically significant between the two performance measures. We also report the number of rankings by average Hausdorff distance and balanced average Hausdorff distance with a Kendall rank correlation coefficient not equal to 1 (Er in Table 2). The Kendall rank correlation coefficient is not equal to 1 when at least one segmentation is misranked in a segmentation set. Twenty segmentation sets were created for each patient so the reported number ranges from 0 to 20 for each patient. With this reported number, we aim to convey a more tangible measure of the ranking capabilities of each of the two performance measures. The statistical analysis was performed using the SciPy package (version 1.5.0) in Python.

## Results

**Fig. 2:**
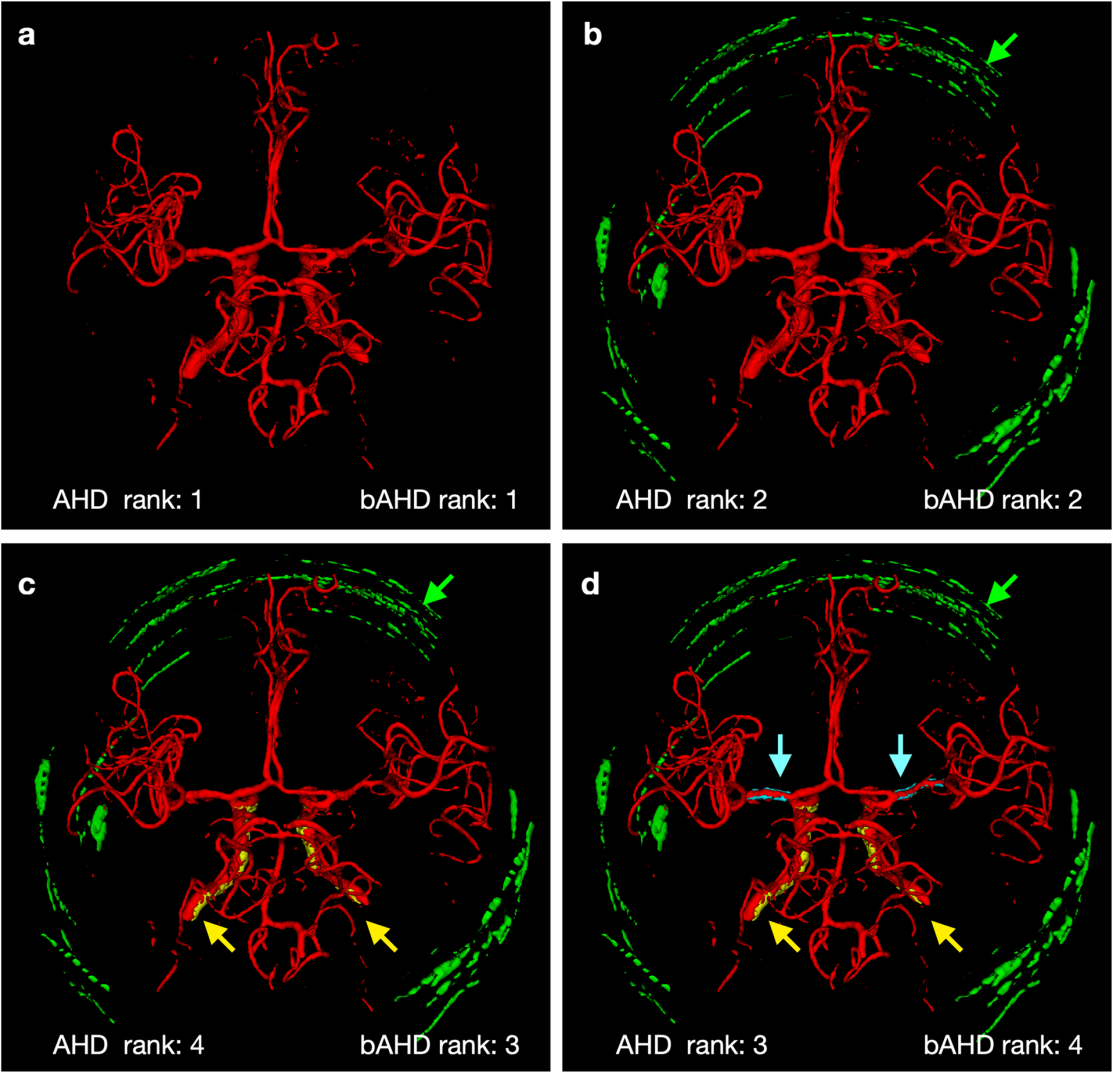
Visual example of the average Hausdorff distance (AHD) ranking error. The ground truth image (**a**) contains no error, (**b**) contains one error, (**c**) two errors, and (**d**) three errors (added errors are indicated with arrows coloured the same as the corresponding error, for a description of the errors see below). AHD misranked (**c** and **d**). In contrast, balanced average Hausdorff distance (bAHD) correctly ranked all three segmentations according to the number of errors contained in the images. Green, error representing false positive voxels in the skull area; yellow, error representing voxels removed bilaterally from the internal carotid arteries; blue, error representing voxels added bilaterally to the M1 segment of the middle cerebral artery

In the pooled analysis of the 200 total rankings, the rankings provided by balanced average Hausdorff distance showed a significantly higher median Kendall’s rank correlation coefficient (1.00) than the rankings provided by average Hausdorff distance (0.89) (*p* = 0.000). An example of rankings produced by each of the two performance measures on a set of segmentations with an increasing number of errors can be found in Table 1. For a complete overview of the averaged results of Kendall’s tau coefficients of the ten patients, see Table 2. For a visual exemplification of the identified ranking error, please refer to Fig. 2.

**Table 1:** Example ranking of a set of segmentations with increasing number of errors by average Hausdorff distance (AHD) and balanced average Hausdorff distance (bAHD)

Segmentations	Count of errors	AHD values	AHD rank	bAHD values	bAHD rank
Ground truth	0	0	1	0	1
E1	1	0.308	2	0.314	2
N1_E1	2	0.455	3	0.467	3
N1_K3_E1	3	9.836	5	23.487	4
N1_H1_K3_E1	4	10.138	7	24.925	5
N1_H1_K3_E1_P991	5	10.111	6	24.928	6
N1_H1_K3_G1_E1_P991	6	10.213	8	25.394	7
N1_H1_K3_G1_E1_P991_M0	7	10.345	9	25.435	8
N1_H1_K3_G1_E1_V2_P991_M0	8	10.638	11	25.613	9
N1_H1_K3_G1_E1_R1_V2_P991_M0	9	10.628	10	25.690	10
N1_H1_K3_G1_E1_R1_V2_P991_C992_M0	10	9.768	4	25.843	11

Values of performance measures (in voxels) are shown with the resulting rankings for one example set. The segmentation ranking of bAHD perfectly correlates with the count of errors in the simulated segmentations. The traditional AHD, however, fails to properly rank the segmentations in line with the number of errors they contain. Error abbreviations are given in the *Segmentations* column. Letters stand for error types and numbers 1, 2, 3 state the intensity levels subtle, moderate and severe, respectively. *K3* False positive errors in the skull area, *C992* Increased radius of the carotid artery (false positive voxels), *M0* Missing M1 segment of the middle cerebral artery (false-negative voxels), *E1* False-positive segmentation of the optical nerve and adjacent fat tissue, *N1* False-positive segmentation of the middle meningeal artery (false-positive voxels), *G1* False-positive segmentation of the sigmoid sinus, *V2* False-negative segmentation of small vessels, *R1* Random voxels added throughout the image (false positives), *H1* False-positive segmentation of the meninges, *P991* Increased radius of the posterior communicating artery (false positives)

**Table 2:** Results of ranking correlation with number of errors for all 10 patients for average Hausdorff distance (AHD) and balanced average Hausdorff distance (bAHD)

**Patient 1**			**Patient 2**			**Patient 3**			**Patient 4**			**Patient 5**		
PM	Tau	Er	PM	Tau	Er	PM	Tau	Er	PM	Tau	Er	PM	Tau	Er
bAHD	1.00	3	bAHD	1.00	4	bAHD	1.00	7	bAHD	1.00	7	bAHD	1.00	6
AHD	0.93	17	AHD	0.93	17	AHD	0.91	18	AHD	0.93	18	AHD	0.87	17
*p* = 0.00039			*p* = 0.00041			*p* = 0.00119			*p* = 0.00018			*p* = 0.00096		
**Patient 6**			**Patient 7**			**Patient 8**			**Patient 9**			**Patient 10**		
PM	Tau	Er	PM	Tau	Er	PM	Tau	Er	PM	Tau	Er	PM	Tau	Er
bAHD	1.00	6	bAHD	1.00	7	bAHD	1.00	5	bAHD	1.00	3	bAHD	1.00	4
AHD	0.84	18	AHD	0.89	18	AHD	0.87	18	AHD	0.89	19	AHD	0.86	19
*p* = 0.00019			*p* = 0.00128			*p* = 0.00064			*p* = 0.00012			*p* = 0.00013		

Median Kendall’s rank correlation coefficients over the 20 sets per patient. For each patient, the rankings produced by bAHD had statistically significantly higher median Kendall rank correlation coefficients compared to rankings of the traditional AHD. This means that the rankings of bAHD have a better agreement with the number of errors in each segmentation and thus bAHD reflects the segmentation quality of cerebral vessel segmentations better than AHD. These results were confirmed by the fact that the bAHD led to less rankings with at least one misranked segmentation compared to AHD as seen in the number of errors column (Er). Approximately three out of four sets of segmentations were ranked perfectly with bAHD where only approximately 1 out of 10 segmentation sets were ranked perfectly with AHD. *PM* Performance measure, *Tau* Average Kendall rank correlation coefficient, *Er* The number of rankings with at least one misranked segmentation within the total number of 20 rankings per patient. The *p* values are obtained by two-sided Wilcoxon signed-rank test comparing the results of 20 sets per patient

In the 200 total rankings analysed, balanced average Hausdorff distance led to 52 rankings with at least one misranked segmentation whilst average Hausdorff distance led to 179 with at least one misranked segmentation. This means that approximately three out of four sets of segmentations ranked by average Hausdorff distance contained a misranked segmentation whereas using balanced average Hausdorff distance only one out of four sets were with a misranked segmentation. The number of misranked segmentation sets can also be found in Table 2. The new performance measure was implemented in the *EvaluateSegmentation* command-line tool that is free to download (https://github.com/Visceral-Project/EvaluateSegmentation).

## Discussion

The average Hausdorff distance is a recommended and widely used performance measure for medical segmentation tasks. In the current paper, we identified a ranking error of this method, making it less suitable to compare segmentation results. We also proposed and validated a new performance measure, balanced average Hausdorff distance, which strongly alleviates this error.

Based on our results, segmentations with lower average Hausdorff distance values do not necessarily correspond to a segmentation of higher quality. Using average Hausdorff distance values to assess segmentation quality may therefore result in an erroneous ranking not reflecting the actual segmentation quality. This ranking error can be explained by the average Hausdorff distance dividing the distance from the ground truth to the segmentation by the number of ground truth voxels whilst dividing the distance from segmentation to the ground truth by the number of voxels in the segmented volume (eq. 2). This leads to an unwanted ranking error in certain situations.

For example, when an error introduces voxels relatively closer to the ground truth than the average distance of the previously introduced errors, the average Hausdorff distance might still decrease, because the increase in *StoG* is proportionally less than the increase of *S*. This results in a lower directed average Hausdorff distance from segmentation to ground truth (*SToG*/*S*). This observation shows that an additional error added to the segmentation might increase *StoG* whilst it simultaneously decreases the average Hausdorff distance indicating an improvement of the segmentation quality. This depends on the distances of the voxels belonging to the error and to the number of voxels contained in the error. Here, although the total distance from segmentation to the ground truth (*StoG*) increases, the denominator corresponding to the number of voxels in the segmentation volume increases as well.

Although the simulated segmentation in Fig. 2d has an additional error compared to Fig. 2c the traditional average Hausdorff distance value of Fig. 2d is lower than that of Fig. 2c resulting in a better rank of Fig. 2d. Therefore, average Hausdorff distance might rank a simulated segmentation containing more errors better than a simulated segmentation with less errors because the denominator changes with the number of voxels in the segmentation. Due to this ranking error, the traditional average Hausdorff distance should be used with caution for rankings and quality assessment of segmentations. This issue was significantly mitigated by the newly proposed balanced average Hausdorff distance, where the *StoG* is divided by the constant number of ground truth voxels instead of the variable number of voxels in the segmentation volume. Applying the new performance measure, the ranking results were strongly improved.

Our study has some limitations. First, even with a balanced average Hausdorff distance, ranking results were not perfect. There are still a few types of errors that increase *StoG* and decrease *GtoS* at the same time. For example, when we simulated false positive single voxels scattered randomly throughout the image volume, we observed increased *StoG* and decreased *GtoS*. This resulted in a lower balanced average Hausdorff distance value, indicating an improved segmentation despite lower quality. Second, the ranking error of average Hausdorff distance could be analysed only for the use case of cerebral vessel segmentation. The ranking error of average Hausdorff distance observed in this study might be more prominent than in other application areas considering that the anatomy and spatial representation of the cerebral arterial tree are relatively complex. Therefore, especially for complex segmentation tasks like vessel tree segmentations (such as in the brain, liver, or heart), balanced average Hausdorff distance should replace the traditional average Hausdorff distance. The ranking properties of both performance measures should be also compared in different application areas to confirm or negate the observations made in this study.

In conclusion, the novel proposed balanced average Hausdorff distance performance measure alleviates the identified ranking error of classic average Hausdorff distance. This makes the balanced average Hausdorff distance more suitable for rankings and quality assessment of segmentations in medical segmentation tasks and should be used instead of the traditional average Hausdorff distance.

## Data Availability

At the current time point, the imaging data cannot be made publicly accessible due to data protection. Researchers interested in the code for error simulation can contact the authors and the data will be made available (either through direct communication or through reference to a public repository).

## Abbreviations

G: Number of voxels in the ground truth
GtoS: Directed average Hausdorff distance from ground truth to segmentation
S: Number of voxels in the segmentation
StoG: Directed average Hausdorff distance from segmentation to ground truth
