# Correction: On the usage of average Hausdorff distance for segmentation performance assessment: hidden error when used for ranking

**DOI:** 10.1186/s41747-022-00309-6

**Published:** 2022-10-31

**Authors:** Orhun Utku Aydin, Abdel Aziz Taha, Adam Hilbert, Ahmed A. Khalil, Ivana Galinovic, Jochen B. Fiebach, Dietmar Frey, Vince Istvan Madai

**Affiliations:** 1 CLAIM - Charité Lab for Artificial Intelligence in Medicine, Charité Universitätsmedizin Berlin, Berlin, Germany; 2 Research Studio Data Science, Research Studios Austria, Salzburg, Austria; 3 Centre for Stroke Research Berlin, Charité Universitätsmedizin Berlin, Berlin, Germany; 4 Department of Neurology, Max Planck Institute for Human Cognitive and Brain Sciences, Leipzig, Germany; 5 Mind, Brain, Body Institute, Berlin School of Mind and Brain, Humboldt-Universität Berlin, Berlin, Germany; 6 School of Computing and Digital Technology, Faculty of Computing, Engineering and the Built Environment, Birmingham City University, Birmingham, UK

**Correction: Eur Radiol Exp 5, 4 (2021)**

**https://doi.org/10.1186/s41747-020-00200-2**

The original article [1] contains a minor notation error in the Methods section of the article regarding the description of the terms “GtoS” and “StoG” in Equation 2 and Equation 3 on page 2 of the PDF version.

The original article states:

- “where GtoS is the directed average Hausdorff distance from ground truth to segmentation, StoG is the directed average Hausdorff distance from segmentation to ground truth, G is the number of voxels in the ground truth, and S is the number of voxels in the segmentation.”

This statement should be disregarded in favour of the following statement:

- “where GtoS is the sum of all minimum distances from all points from the ground truth to segmentation, StoG is the sum of all minimum distances from all points from the segmentation to ground truth, G is the number of voxels in the ground truth, and S is the number of voxels in the segmentation.”

This notation error is also present in the Abbreviations section of the article and should be corrected as well.

The original article states:

- “GtoS: Directed average Hausdorff distance from ground truth to segmentation
  StoG: Directed average Hausdorff distance from segmentation to ground truth”

This statement should be disregarded in favour of the following statement:

- “GtoS: the sum of all minimum distances from all points from the ground truth to segmentation
  StoG: the sum of all minimum distances from all points from the segmentation to ground truth”

The authors would importantly like to note that this notation error does not have an impact on the results of our paper.

The authors also note that the terms StoG and GtoS are used correctly in other sections of the article and the notation error only affects the Methods and Abbreviations sections.
